# 改进QuEChERS法结合超高效液相色谱-串联质谱法同时测定冷冻食品中7种季铵盐化合物

**DOI:** 10.3724/SP.J.1123.2022.05008

**Published:** 2023-03-08

**Authors:** Bolin LIU, Ziwei ZHAO, Ziyue ZHAN, Yanyu DAI, Gang DING, Ji’an XIE, Xiangchuan XU, Hongtao KONG

**Affiliations:** 1.安徽省疾病预防控制中心, 安徽 合肥 230601; 1. Anhui Provincial Center for Disease Control and Prevention, Hefei 230601, China; 2.安徽启威生物科技有限公司, 安徽 合肥 230000; 2. Anhui Kiwi Biotechnology Company Limited, Hefei 230000, China

**Keywords:** QuEChERS, 超高效液相色谱-串联质谱, 季铵盐化合物, 冷冻食品, QuEChERS, ultra performance liquid chromatography-tandem mass spectrometry (UPLC-MS/MS), quaternary ammonium compounds (QACs), frozen food

## Abstract

近年来,季铵盐类消毒剂的广泛使用,导致其残留在食品或环境中,并经食物链进入人体,引起呼吸系统和生殖系统的不良反应,威胁人类身体健康。为此,建立了QuEChERS-超高效液相色谱-串联质谱(QuEChERS-UPLC-MS/MS)同时测定冷冻食品中6种常规季铵盐化合物和1种新型季铵盐化合物残留的分析方法。对样品前处理方法进行改良,色谱条件进行优化,样品经20 mL 90%甲醇水溶液(含0.5%甲酸)涡旋提取20 min后,超声10 min, 10000 r/min离心10 min,取1 mL上清液放入装有100 mg PSA净化剂的15 mL离心管中净化,涡旋1 min后,10000 r/min离心5 min,取上清液直接上机测定。以甲醇与5 mmol/L乙酸铵溶液作为流动相进行梯度洗脱,采用ACQUITY UPLC BEH C_8_色谱柱(50 mm×2.1 mm, 1.7 μm)分离,在ESI^+^模式,多反应监测(MRM)方式下采集数据,基质匹配曲线外标法定量。结果表明,7种待测物在C_8_色谱柱上完全基线分离,在0.1~100.0 μg/L的范围内线性关系良好,相关系数(*r*^2^)为0.9971~0.9983,该方法的检出限(LOD)为0.5~1.0 μg/kg,定量限(LOQ)为1.5~3.0 μg/kg。以阴性三文鱼、鸡肉为基质样品,在低、中、高3个水平(3.0、10.0、100.0 μg/kg)下进行加标回收试验,各待测物的平均回收率为65.4%~101%,相对标准偏差(RSD)为0.64%~16.8%。该检测方法灵敏度高,选择性强,稳定性好,结果准确可靠,适用于冷冻食品中7种季铵盐化合物的批量快速测定,同时,弥补了新型季铵盐化合物检测方法的空白。

## Abstract (en)

Quaternary ammonium compounds (QACs) are a class of cationic surfactants that can be used as the main active ingredient of disinfectants. The increased use of QACs is concerning as exposure from inhalation or ingestion to these compounds that has been associated with adverse effects on the reproductive and respiratory systems. Humans are exposed to QACs primarily by food consumption and inhalation of air. QAC residues pose significant threats to public health. Given the importance of assessing potential residue levels for QACs in food, therefore, a method was developed for the simultaneous detection of six common QACs and one emerging QAC (Ephemora) in frozen food by ultra performance liquid chromatography-tandem mass spectrometry (UPLC-MS/MS) coupled with the modified QuEChERS method. The main factors governing the response, recovery, and sensitivity of the method, including extraction solvents, types and dosages of adsorbents, apparatus conditions, and mobile phases, were optimized in the course of sample pretreatment and instrument analysis. QAC residues in frozen food were extracted using 20 mL methanol-water (90∶10, containing 0.5% formic acid) for 20 min by the vortex shock method. The mixture was ultrasonicated for 10 min and centrifuged at 10000 r/min for 10 min. A 1-mL aliquot of the supernatant was transferred to a new tube and purified using 100 mg of PSA adsorbents. After mixing and centrifugation at 10000 r/min for 5 min, the purified solution was analyzed. Target analytes were separated on an ACQUITY UPLC BEH C_8_ chromatographic column (50 mm×2.1 mm, 1.7 μm) at a column temperature of 40 ℃ and a flow rate of 0.3 mL/min. The injection volume was 1 μL. Gradient elution was performed using methanol and 5 mmol/L ammonium acetate solution as the mobile phases. Multiple reaction monitoring (MRM) was conducted in the positive electrospray ionization (ESI^+^) mode. The matrix-matched external standard method was used to quantify seven QACs. The optimized chromatography-based method completely separated the seven analytes. Good linear relationships were obtained for the seven QACs in the range of 0.1-100.0 ng/mL. The correlation coefficient (*r*^2^) ranged from 0.9971 to 0.9983. The limits of detection and limits of quantification ranged from 0.5 to 1.0 μg/kg and 1.5 to 3.0 μg/kg, respectively. Accuracy and precision were determined by spiking salmon and chicken samples with 3.0, 10.0, and 100.0 μg/kg of analytes, in compliance with the current legislation, with six replicates per determination. The average recoveries of the seven QACs ranged from 65.4% to 101%. The relative standard deviations (RSDs) were between 0.64% and 16.8%. Matrix effects of the analytes were between -27.5% and 33.4% in salmon and chicken samples after purifying using PSA. The developed method was applied to the determination of seven QACs in rural samples. QACs were detected in only one sample; the level did not exceed European Food Safety Authority specified residue limit standards. The detection method has high sensitivity, good selectivity and stability, and the results are accurate and reliable. It is suitable for the simultaneous rapid determination of seven QAC residues in frozen food. The results provide valuable information for future risk assessment studies targeting this class of compounds.

季铵盐化合物(quaternary ammonium compounds, QACs)是一类由带正电荷的季氨基和一个或两个长烷基链组成的有机物^[1]^,季铵盐化合物作为消毒剂、抗菌剂和表面活性剂的活性成分,广泛应用于清洁与个人护理产品^[1,2]^、医疗卫生^[3]^和食品工业领域^[4]^,常见的季铵盐化合物包括烷基二甲基苄基氯化铵类化合物(benzylalkyldimethyl ammonium compounds, BACs)、二癸基二甲基氯化铵类化合物(dialkyldimethyl ammonium compounds, DDACs)和烷基三甲基氯化铵类化合物(alkyltrimethyl ammonium compounds, ATMACs)^[5]^。美国允许这些常规季铵盐化合物大规模生产和使用,我国GB/T 26369-2020《季铵盐类消毒剂卫生要求》中规定季铵盐化合物的使用范围包括物体与医疗器械表面、织物、外科手消毒、卫生手消毒、皮肤与黏膜、食品加工设备与器皿的消毒^[6]^。研究表明,季铵盐化合物可有效灭活包膜病毒^[7,8]^, 2020年,美国环境保护署(EPA)列出的430种可以杀灭新型冠状病毒的消毒剂产品中,有216种含有季铵盐活性成分^[9]^。因此,近年来大量的消毒剂被用于室内外环境的消毒,以限制疾病的传播^[2]^。此外,包括我国在内的许多国家在冷链食品或外包装表面检测到新冠病毒的核酸^[10]^,因此冷链食品在生产经营过程中需要进行消毒工作。而季铵盐类消毒剂可用于物体表面、低温冷藏物体及冷冻物体表面的消毒,这使得季铵盐类消毒剂在冷链食品中广泛应用。

然而,季铵盐类消毒剂的大规模生产和使用,会导致其残留在环境中,地表水^[11]^、河流底泥^[1,12,13]^和土壤^[14]^均被报道检出季铵盐化合物,我国城市污水污泥中检出ATMACs与BACs,其含量分别为0.38~293 μg/g与0.09~191 μg/g^[15]^。牛奶^[16]^、冷冻饮品^[4]^等食品中也检出季铵盐化合物。室内粉尘中季铵盐化合物含量达到了1.95~531 μg/g^[17]^,人体血液中QACs含量大幅增加^[5]^。可见,季铵盐化合物残留已通过饮食、空气环境和皮肤吸收进入人体。毒理学研究表明,季铵盐化合物对水生生物具有较强的毒性^[18]^,对生殖系统和呼吸系统有不良反应^[17]^。长期暴露于季铵盐,会引起哮喘病、皮肤过敏性反应疾病^[19,20]^,对人类身体健康产生风险^[21]^。为此,欧洲食品安全局(EFSA)规定食品中季铵盐化合物残留限量为0.1 mg/kg^[4]^,而我国还没有制定食品中季铵盐化合物残留限量标准和标准检测方法,因此建立一种简便快捷、灵敏、选择性和准确度高的检测方法测定冷冻食品中季铵盐化合物具有重要意义。

目前,针对季铵盐化合物的检测方法主要有离子色谱法^[22]^、气相色谱-质谱联用法(GC-MS)^[23]^、高效液相色谱法(HPLC)^[24⇓⇓⇓-28]^与高效液相色谱-串联质谱法(HPLC-MS/MS)^[4,29⇓-31]^,这些方法满足了消毒剂、食品等基质中常规季铵盐化合物的检测要求。随着对季铵盐化合物杀菌效果的研究,新型季铵盐化合物不断被合成,并投入生产使用。2021年,Zhang等^[32]^合成了一种新型季铵盐化合物2-甲基-1-十二烷基硫醇丙酸氯化胆碱(Ephemora),其具有与常规季铵盐类似的结构式,可以用作消毒剂的活性成分,且易降解、绿色环保,但目前缺少这类新型季铵盐化合物的检测方法。因超高效液相色谱-串联质谱法(UPLC-MS/MS)具有选择性、灵敏度和分析效率高等优势,质谱方法能够提供待测物碎片离子信息,降低了假阳性率,成为季铵盐化合物检测分析的主要方法^[31]^。本研究在文献基础上,优化色谱与质谱条件,改进提取与净化方法,降低基质效应,以冷冻三文鱼、鸡肉为基质样品,采用基质匹配外标法定量,建立了改进QuEChERS法前处理样品、UPLC-MS/MS快速定量测定冷冻食品中6种常规季铵盐与1种新型季铵盐共7种化合物的分析方法,为制定冷冻食品中季铵盐化合物残留限量标准提供基础数据,为新型季铵盐类消毒剂产品上市后的监测与监管提供高效、便捷、准确可靠的技术支持。

## 1 实验部分

### 1.1 仪器与试剂

ACQUITY^TM^ UPLC超高效液相色谱-Xevo TQ质谱仪、ACQUITY^TM^ UPLC BEH C_8_色谱柱(50 mm×2.1 mm, 1.7 μm)、保护柱(5 mm×2.1 mm, 1.7 μm)(美国Waters公司);多点振荡器(德国Heidolph公司); Milli-Q超纯水制备仪(美国Millipore公司); Legend Mach 1.6R高速冷冻离心机(美国Thermo Fisher公司)。

甲醇、乙腈与甲酸(均为色谱纯,德国Merck公司);乙酸铵(色谱纯,美国Sigma-Aldrich公司); *N*-丙基乙二胺(PSA, 40 μm,美国Agilent公司);标准品苄基十二烷基二甲基氯化铵(benzyldimethyldodecylammonium chloride, C12-BAC,纯度99%)、苄基十四烷基二甲基氯化铵(benzyldimethyltetradecylammonium chloride, C14-BAC,纯度98%)、苄基十六烷基二甲基氯化铵(benzyldimethylhexadecylammonium chloride, C16-BAC,纯度95%)、十二烷基三甲基溴化铵(dodecyl trimethyl ammonium bromide, C12-ATMAC,纯度99%)、双十烷基二甲基氯化铵(didecyldimethylammonium chloride, C10-DDAC,纯度95%)与度米芬(domiphen bromide,纯度97%)购自美国Sigma-Aldrich公司;Ephemora(纯度99.0%)由安徽启威生物科技有限公司提供。

### 1.2 标准溶液的配制

分别称取C12-BAC、C14-BAC、C16-BAC、C12-ATMAC、C10-DDAC、度米芬与Ephemora各10 mg(准确至0.01 mg),分别用甲醇溶解并定容至10 mL,配制1.0 mg/mL标准储备溶液,-20 ℃下密封保存。

分别准确吸取上述标准储备液适量于同一容量瓶中,加90%甲醇水溶液(含0.5%甲酸)稀释至刻度,得到10 μg/mL的混合标准中间液;吸取上述混合标准使用液适量于10 mL容量瓶中,加90%甲醇水溶液(含0.5%甲酸)稀释至刻度,得到100 ng/mL的混合标准使用液;密封后4 ℃保存。

分别准确移取上述混合标准使用液(100 ng/mL)5、10、50、100、500、1000与5000 μL到10 mL容量瓶中,用90%甲醇水溶液(含0.5%甲酸)定容至刻度,配制0.05、0.1、0.5、1.0、5.0、10.0与50.0 ng/mL的系列标准溶液,现配现用。

### 1.3 样品前处理

冷冻三文鱼、鸡肉样品于室温化冻后,取500 g可食用的肌肉部分,均质机打碎混匀,称取1.0 g(精确到0.001 g)均匀样品于50 mL离心管中,加入18 mL 90%甲醇水溶液(含0.5%甲酸)提取液,涡旋振荡20 min,超声提取10 min,以10000 r/min离心10 min,上清液转移至50 mL离心管中,加90%甲醇水溶液(含0.5%甲酸)定容至20 mL,混匀后,移取1 mL上清液置于装有100 mg PSA净化剂的15 mL离心管中,涡旋1 min后,10000 r/min离心5 min,取上层净化液直接用UPLC-MS/MS进样测定。

### 1.4 分析条件

#### 1.4.1 色谱条件

色谱柱:UPLC BEH C_8_柱(50 mm×2.1mm, 1.7 μm),保护柱(5 mm×2.1 mm, 1.7 μm);柱温:40 ℃;样品室温度:10 ℃;流速:0.3 mL/min;进样量:1 μL;流动相A: 5 mmol/L乙酸铵水溶液;流动相B:甲醇。梯度洗脱程序:0~3.0 min, 50%A~20%A; 3.0~5.0 min, 20%A; 5.0~5.1 min, 20%A~50%A; 5.1~7.1 min, 50%A。

#### 1.4.2 质谱条件

离子源:电喷雾电离(ESI^+^)源;毛细管电压:3.50 kV;离子源温度:150 ℃;脱溶剂气温度:500 ℃;脱溶剂气流量:800 L/h;碰撞气流量:0.12 mL/min;多反应监测(MRM)模式。待测物的母离子、子离子及对应的碰撞能量、锥孔电压等质谱参数见表1。

**表1:** 7种季铵盐化合物的质谱参数

Analyte	Abbreviation	Mode	t_R_/ min	Parent ion (m/z)	Product ions (m/z)	Collision energies/eV	Cone voltage/V
Dodecyl trimethyl ammonium bromide (十二烷基三甲基溴化铵)	C12-ATMAC	[M-Br]^+^	2.70	228.2	60.0^*^/57.1/71.0	24/26/24	64
Benzyldimethyldodecylammonium chloride (苄基十二烷基二甲基氯化铵)	C12-BAC	[M-Cl]^+^	3.17	304.2	91.0^*^/58.0/212.2	30/26/22	56
Domiphen bromide (度米芬)		[M-Br]^+^	3.55	334.2	72.0^*^/58.0/166.1	30/30/24	68
Benzyldimethyltetradecylammonium chloride (苄基十四烷基二甲基氯化铵)	C14-BAC	[M-Cl]^+^	3.77	332.2	91.0^*^/58.0/240.3	30/26/24	60
2-Methyl-1-dodecanethiol propionate choline chloride (2-甲基-1-十二烷基硫醇丙酸氯化胆碱)	Ephemora	[M-Cl]^+^	4.03	374.2	113.0^*^/215.2/315.2	24/26/18	56
Didecyldimethylammonium chloride (双十烷基二甲基氯化铵)	C10-DDAC	[M-Cl]^+^	4.26	326.3	186.2^*^/57.1	32/28	76
Benzyldimethylhexadecylammonium chloride (苄基十六烷基二甲基氯化铵)	C16-BAC	[M-Cl]^+^	4.44	360.3	91.0^*^/58.0/268.3	30/28/24	64

* Quantitative ion.

## 2 结果与讨论

### 2.1 质谱条件的优化

选择的7种季铵盐化合物结构式中均含有正电荷的N和负电荷的Cl或Br,在水溶液中易离解成离子,以[M-Cl]^+^或[M-Br]^+^的形式存在,采用电喷雾离子源易获得高丰度的分子离子。配制500 ng/mL标准溶液,采用直接进样方式,ESI^+^模式下进行全扫描,确定待测物的分子离子,以分子离子峰作为母离子进行子离子扫描,获得各待测物的子离子。以新型季铵盐化合物Ephemora为例,其相对分子质量为410.1,分子离子峰[M-Cl]^+^ (*m/z* 374.2),结构式如图1所示,酯基和烷基易发生断裂,产生丰度较强的*m/z* 113.0碎片。按照欧盟委员会执行条例(EU) 2021/808的要求,采用低分辨质谱分析残留时,至少满足1个母离子和两子离子进行确证的要求,本实验选择离子丰度最强的子离子作为定量离子,两个离子丰度较强的子离子作为定性离子。使用Waters TQ MS自动调谐分析功能,调节锥孔电压,优化子离子的碰撞能量等质谱参数,经优化后的参数如表1所示。

**图1:**
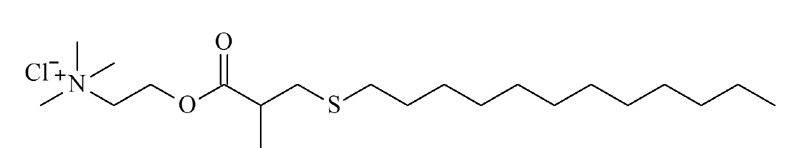
新型季铵盐化合物的分子结构式

### 2.2 色谱条件的优化

实验比较了Waters BEH C_18_ (100 mm×2.1 mm, 1.7 μm)、Waters BEH T3 (100 mm×2.1 mm, 1.8 μm)、Waters BEH C_18_ (50 mm×2.1 mm, 1.7 μm)和Waters BEH C_8_ (50 mm×2.1 mm, 1.7 μm)色谱柱对7种待测物的分离、响应值和峰形的影响,实验表明,7种待测物在BEH C_18_色谱柱上无响应值,无法分离出色谱峰,这与待测物含有较长的碳链有关,容易被C_18_色谱柱中的填料吸附,较难洗脱^[4]^。采用BEH T3色谱柱时,待测物色谱峰严重拖尾,无法获得满意的峰形;采用BEH C_8_色谱柱时,待测物的峰形对称尖锐,响应值较高,7种待测物达到基线分离。故选择BEH C_8_色谱柱作为分析柱。

实验考察了0.1%(v/v)甲酸水溶液-乙腈、0.1%(v/v)甲酸水溶液-甲醇、5 mmol/L乙酸铵溶液-乙腈与5 mmol/L乙酸铵溶液-甲醇等流动相对色谱分离及灵敏度的影响。实验发现,采用甲醇为有机相时,7种季铵盐化合物的响应值均高于乙腈。在水相中加入少量的甲酸或乙酸铵,能改善待测物的峰形,增加灵敏度,且5 mmol/L乙酸铵溶液的灵敏度高于0.1%(v/v)甲酸水溶液。确定甲醇-5 mmol/L乙酸铵溶液为流动相,优化梯度洗脱程序,待测物的峰形尖锐对称、灵敏度高。

### 2.3 样品前处理条件的优化

#### 2.3.1 提取溶剂的选择

标准方法^[33]^采用酸化甲醇提取消毒剂中C12-BAC、C14-BAC、C16-BAC、C12-ATMAC、C10-DDAC和度米芬。针对食品中的季铵盐化合物,文献报道的提取溶剂有乙腈^[16]^、乙腈-乙酸乙酯(50∶50, v/v)混合液^[31]^等。本实验比较了甲醇、乙腈、酸化甲醇、酸化乙腈、甲醇-水溶液、乙腈-水溶液等不同提取溶剂的提取效果,以阴性三文鱼为基质样品,添加25.0 μg/kg标准液进行加标回收。结果显示,提取溶剂中含有甲醇时7种待测物的响应值高,回收率优于乙腈。进一步比较不同体积分数的甲醇水溶液(10%~90%)后发现,随着甲醇体积分数的增加,待测物的峰面积逐渐增加,甲醇体积分数为90%时7种待测物的响应值最高。同时,正离子模式下,甲酸提供的H^+^,可提高离子化效率。在90%甲醇水溶液加入不同体积分数的甲酸(0.1%~0.5%)后发现,随着甲酸体积分数的增加,待测物的灵敏度增加,甲酸体积分数为0.5%时灵敏度与回收率最高。因此,确定90%甲醇水溶液(含0.5%甲酸)为提取溶剂。

#### 2.3.2 净化剂及含量的选择

将提取液经4 ℃下10000 r/min离心5 min,取上清液直接进样,发现7种季铵盐化合物的加标回收率为107%~132%,除C16-BAC的回收率为107%之外,其余化合物的回收率均大于120%,超出60%~120%的范围,且未经过净化的进样液颜色较深,高速离心无法除去色素等杂质,存在共提物干扰响应,同时易造成污染,影响仪器与色谱柱的寿命。针对复杂食品基质的净化处理,有固相萃取柱法、QuEChERS法、分散固相萃取法(dSPE)、PRiME HLB柱一步净化法等,而QuEChERS法以快速、简单、廉价、有效、安全的优势被广泛应用^[34]^。常见的净化剂有PSA、C_18_和石墨化炭黑(GCB)。针对季铵盐化合物的净化,已发现C_18_和GCB会吸附季铵盐,导致回收率降低,仅采用PSA净化时,回收率高^[29]^。本实验考察了不同PSA用量(50、75、100、150 mg)下的净化效果。以阴性三文鱼为基质样品,添加7种季铵盐混合标准液,使其含量为25.0 μg/kg,按照1.3节样品前处理方法提取后上机测定,比较加标回收率。结果表明,提取液未经PSA净化,C14-BAC的回收率为113%,其余待测物的加标回收率均大于120%;经50 mg PSA净化后,各待测物的回收率为105%~116%,基质增强效应得到改善;增加PSA用量,各待测物的回收率有所降低;当PSA用量增加到100 mg时,各待测物的回收率为94.0%~109%,满足GB/T 27417-2017的相关要求(回收率为60%~120%)。综合考虑净化效果和待测物回收率,选用100 mg PSA为最佳净化剂。

### 2.4 基质效应

选用冷冻阴性三文鱼与鸡肉为基质样品,按1.3节方法处理,获得空白基质液,用该空白基质液配制系列基质匹配标准溶液,同时,采用90%甲醇水溶液(含0.5%甲酸)溶剂配制系列标准溶液。根据文献^[35]^报道的方法,采用公式ME=(基质匹配标准曲线的斜率/溶剂标准溶液曲线的斜率-1)×100%来计算7种季铵盐化合物的基质效应,以评价食品基质中共提物对季铵盐化合物响应值的影响。按照|ME|> 50%为强基质效应,20%<|ME|<50%为中等基质效应,|ME|<20%为弱基质效应的判定依据,结果显示,三文鱼样品对Ephemora具有基质抑制效应(ME为-4.83%),对C12-BAC、C14-BAC、C16-BAC、C12-ATMAC、C10-DDAC和度米芬均具有基质增强效应,其ME值分别为11.9%、19%、33.4%、18.3%、2.92%和8.29%, C16-BAC在三文鱼样品基质中为中等基质效应,其余6种均为弱基质效应。鸡肉样品对C12-ATMAC与C16-BAC具有基质增强效应,对C12-BAC、C14-BAC、C10-DDAC和度米芬具有基质抑制效应,鸡肉样品中7种季铵盐化合物的ME范围为-27.5%~14.9%,除Ephemora为中等基质效应外,C12-BAC、C14-BAC、C16-BAC、C12-ATMAC、C10-DDAC和度米芬均为弱基质效应。7种季铵盐化合物在实际样品中的基质效应大小如图2所示。可见不同的食品基质样品对7种季铵盐化合物有基质效应的影响,在实际样品的检测中,本实验采用阴性空白基质匹配标准曲线定量进一步补偿基质效应的影响。

**图 2:**
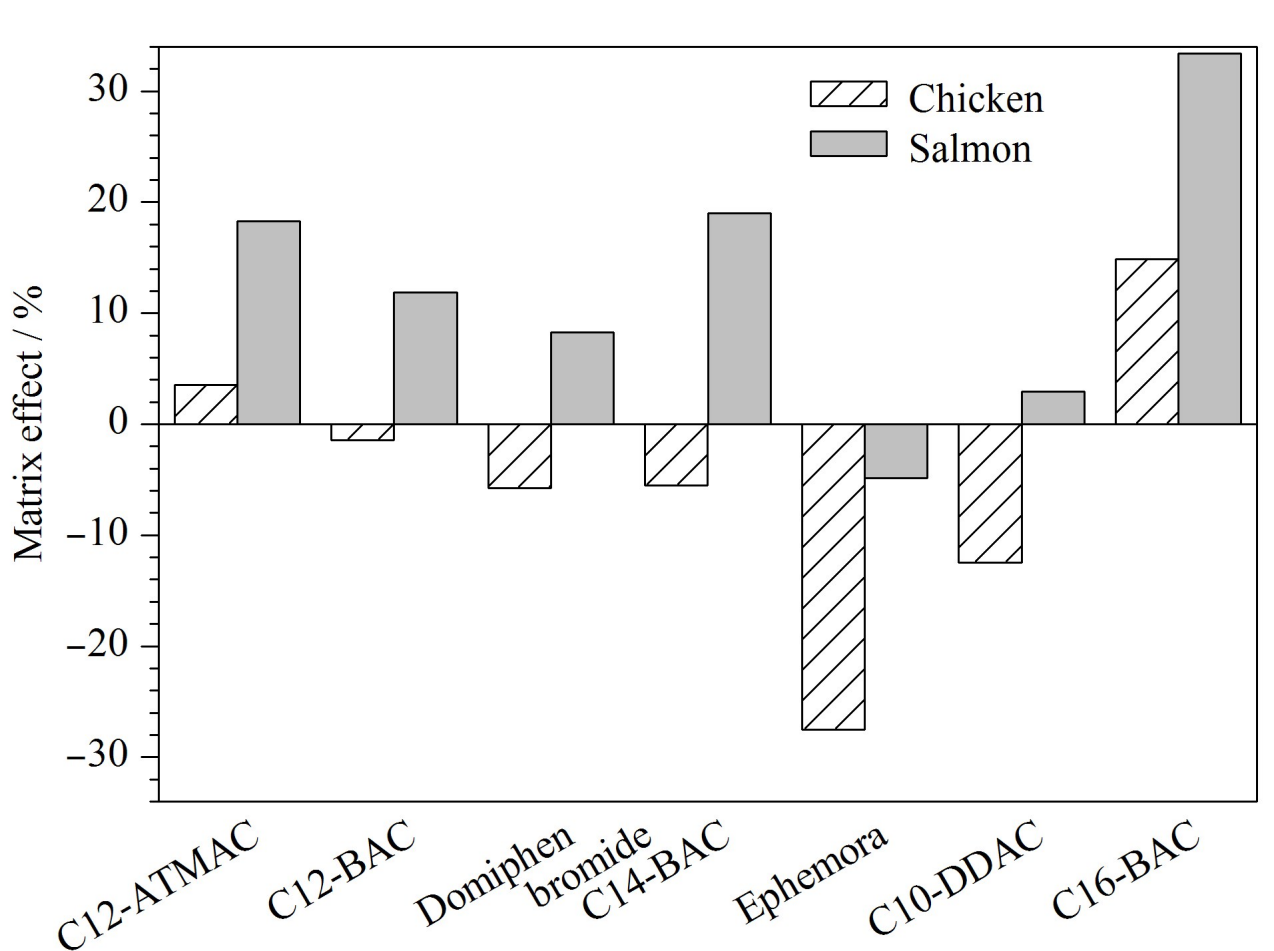
鸡肉、三文鱼基质样品中7种季铵盐化合物的基质效应

### 2.5 方法学验证

#### 2.5.1 线性关系、检出限和定量限

取适量的7种季铵盐标准使用液,采用阴性三文鱼与鸡肉样品的基质液配制0.1、0.5、1.0、5.0、10.0、50.0、100 ng/mL的系列标准溶液,优化的仪器条件下上机测定,以待测物峰面积(*y*)为纵坐标,质量浓度(*x*, ng/mL)为横坐标,绘制基质匹配标准曲线,外标法定量。7种季铵盐化合物在0.1~100 ng/mL范围内呈良好的线性关系,线性相关系数(*r*^2^)范围为0.9971~0.9983(见表2)。

**表2:** 7种季铵盐化合物的线性范围、线性方程、相关系数、检出限及定量限

Analyte	Linear range/(ng/mL)	Linear equation	r^2^	LOD/(μg/kg)	LOQ/(μg/kg)
C12-BAC	0.1-100	y=2.99×10^3^x+1.75×10^2^	0.9971	1.0	3.0
C14-BAC	0.1-100	y=5.49×10^3^x+2.05×10^2^	0.9973	1.0	3.0
C16-BAC	0.1-100	y=3.85×10^3^x+2.30×10	0.9972	1.0	3.0
C12-ATMAC	0.1-100	y=1.70×10^3^x+1.07×10^2^	0.9976	1.0	3.0
C10-DDAC	0.1-100	y=1.43×10^3^x+6.45×10	0.9983	1.0	3.0
Domiphen bromide	0.1-100	y=1.89×10^3^x+1.88×10	0.9979	1.0	3.0
Ephemora	0.1-100	y=4.65×10^3^x+6.50	0.9977	0.5	1.5

*y*: peak area; *x*: mass concentration, ng/mL.

选择阴性三文鱼基质样品,添加适量浓度的7种混合标准溶液,按照上述1.3节方法进行样品前处理,上机测定,分别以3倍信噪比与10倍信噪比对应的含量为检出限(LOD)与定量限(LOQ),该方法的LOD为0.5~1.0 μg/kg, LOQ为1.5~3.0 μg/kg(见表2)。

#### 2.5.2 准确度与精密度

选择阴性的基质样品进行加标回收试验,以验证方法的准确度和精密度。标准GB/T 27404-2008规定,已制定食品最大残留限量(MRL)的物质,选择浓度水平为方法定量限、MRL值与合适浓度点进行加标,未制定MRL的物质,选择浓度水平为方法定量限、常见限量值与合适浓度点进行加标回收试验。目前我国还没有制定食品中季铵盐化合物的最大残留限量,欧盟规定食品中季铵盐化合物残留限量为0.1 mg/kg。本研究依据上述相关规定进行加标试验,具体方法如下:准确称取1.0 g阴性样品,添加不同浓度水平(低、中、高)的7种季铵盐混合标准液,使其添加量分别为3.0(Ephemora为1.50)、10.0与100.0 μg/kg,按照1.3节方法处理样品后,上机测定,每个添加水平平行测定6次,结果如表3所示,平均回收率范围为65.4%~101%,均在60%~120%范围内,相对标准偏差(RSD)范围为0.64%~16.8%,符合GB/T 27417-2017的相关要求。

**表3:** 冷冻鸡肉、三文鱼样品中7种季铵盐化合物的 加标回收率和相对标准偏差(*n*=6)

Analyte	Spiked level/ (μg/kg)	Recoveries/%			RSDs/%		
		Chicken	Salmon		Chicken	Salmon	
C12-BAC	3.00	93.5	80.1		16.8	5.14	
	10.0	86.3	79.4		10.2	10.3	
	100	84.6	86.2		9.59	8.87	
C14-BAC	3.00	95.5	101		7.57	8.12	
	10.0	88.3	77.8		6.31	3.92	
	100	84.1	94.7		5.85	6.25	
C16-BAC	3.00	86.0	92.0		9.97	9.97	
	10.0	85.4	73.8		6.28	5.34	
	100	81.6	82.1		6.32	6.36	
C12-ATMAC	3.00	88.5	82.4		7.83	11.1	
	10.0	76.7	72.9		10.2	6.70	
	100	80.7	81.0		5.32	6.78	
C10-DDAC	3.00	88.0	65.4		9.97	10.2	
	10.0	77.6	74.3		1.43	3.68	
	100	76.9	82.1		3.06	11.6	
Domiphen	3.00	87.7	86.5		10.6	2.93	
bromide	10.0	88.0	79.6		4.26	13.7	
	100	84.9	84.8		4.03	1.05	
Ephemora	1.50	74.5	83.3		14.5	5.73	
	10.0	68.1	69.7		0.64	1.51	
	100	67.9	81.0		2.23	5.29	

### 2.6 实际样品的测定

采用建立的分析方法对从超市采集的15份冷冻水产品进行检测,包括冻罗非鱼片、冻三文鱼、冻南美对虾等品种。仅1份样品中检出季铵盐化合物,含量值为27.6 μg/kg,未超出EFSA规定的食品中季铵盐化合物残留限量标准。冷冻水产品中检出季铵盐化合物有可能来自环境的污染或消毒处理时的残留。

## 3 结论

基于QuEChERS法,改进样品前处理与色谱-质谱条件,通过一次性前处理对6种常见季铵盐化合物和1种新型季铵盐化合物进行提取、净化,结合超高效液相色谱-串联质谱进行测定,可在5.0 min内完成7种季铵盐化合物的快速定量检测。该方法简便快捷,灵敏度高,选择性强,基质效应低,适用于批量冷冻食品中季铵盐化合物的测定,同时,该方法弥补了新型季铵盐化合物Ephemora检测方法的空白,为新型季铵盐消毒剂产品的监管提供了检测技术,为制定我国食品中季铵盐化合物的最大残留限量提供了数据支持。
